# Hajdu-Cheney Syndrome With Coexisting Rheumatoid Arthritis: A Diagnostic Challenge

**DOI:** 10.7759/cureus.101541

**Published:** 2026-01-14

**Authors:** Mehadi Hasan, Sanjida Sharmin, Abdullah Al Hossain Nidul

**Affiliations:** 1 Internal Medicine, Evercare Hospital Dhaka, Dhaka, BGD; 2 Obstetrics and Gynaecology, Dhaka Medical College Hospital, Dhaka, BGD; 3 Critical Care, Dhaka National Medical Institute Hospital, Dhaka, BGD

**Keywords:** acro-osteolysis, hajdu-cheney syndrome, notch2 gene, osteoporosis, rheumatoid arthritis

## Abstract

We report a case of a 29-year-old female with clinical features consistent with a very rare connective tissue disorder known as Hajdu-Cheney syndrome (HCS), with coexisting inflammatory arthritis suggestive of rheumatoid arthritis. HCS primarily affects the skeletal system and demonstrates a broad spectrum of clinical presentations, including possible systemic manifestations. It is thought that a change in the NOTCH2 gene plays a role in HCS. Genetic confirmation is not always available, and diagnosis often relies on clinical and radiological findings. This case highlights the diagnostic considerations and management challenges encountered when HCS is suspected, particularly in the presence of overlapping inflammatory joint disease.

## Introduction

Hajdu-Cheney syndrome (HCS) is a disorder characterized by acro-osteolysis, short stature, severe osteoporosis, specific characteristics of the craniofacial region, and wormian bones. Additional features may include a range of neurological manifestations with cardiovascular anomalies and polycystic kidney disease. Despite being an autosomal dominant condition, many cases are reported as isolated instances [1]. The diagnosis is made by the radiological findings and the clinical presentation. This case is presented to highlight the diagnostic complexity of HCS. It also emphasizes the challenges that arise when inflammatory joint disease is present. Careful interpretation and individualized clinical decision-making are essential in such rare disorders.

## Case presentation

A 29-year-old female presented with complaints of progressive shortening of fingers, recurrent low-trauma fractures, premature loss of permanent teeth, and multiple joint pain for the last 17 years. She was the first offspring of nonconsanguineous parents and was born full term with an uneventful normal delivery. Up to 12 years of age, she was growing normally. Then she noticed a gradual shortening and widening of her fingers with nail changes. Due to these changes, she had difficulty gripping objects. There were several episodes of low-trauma fractures, as well as atraumatic fractures of her forearm and hand. She also experienced premature loss of her teeth. Her permanent teeth had not erupted properly. Teeth were misaligned and irregular in shape. She was also having pain in the different joints of her hands. Pain initially started in the right wrist joint and subsequently involved the small joints of the hands, particularly the distal interphalangeal (DIP) joints. There was no morning stiffness, and the movements of the joints were normal. Her mother had a similar illness with significant shortening of fingers and deformities of hands and feet. She also had several episodes of atraumatic vertebral fractures. Her deformities were so severe that she was unable to perform routine activities. She had one younger brother, who was 24 years old and living a healthy life.

On general examination, she was short-statured with characteristic craniofacial abnormalities, had downward slanting of the upper eyelid, arched eyebrows, a long philtrum, hirsutism, micrognathia, retrognathism, a prominent occiput, a soft posterior fontanelle with the feeling of multiple small bones along the suture lines, and mild kyphosis. There was an absence of multiple teeth. The remaining teeth were misaligned with multiple dental implantations.

**Figure 1:**
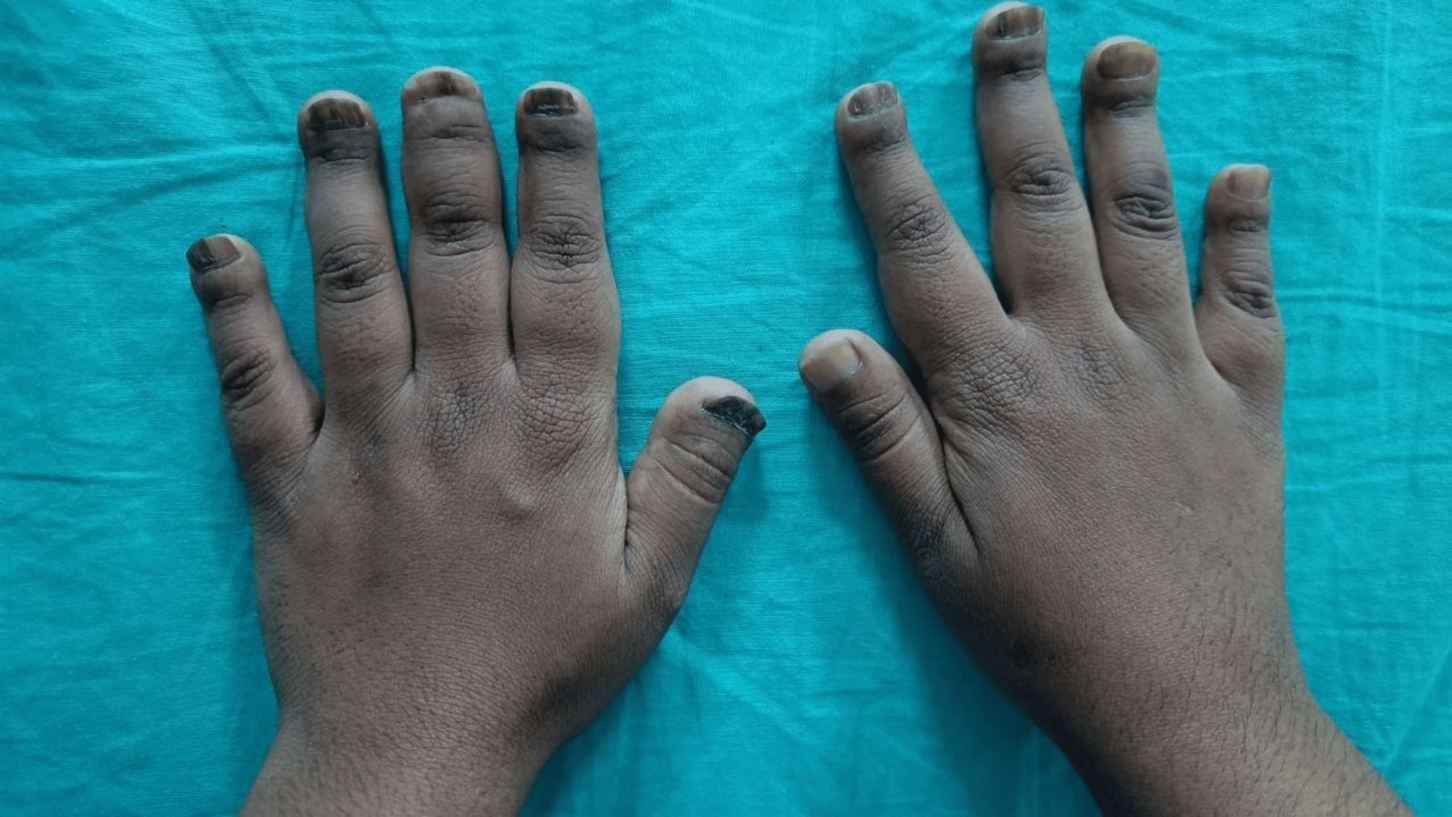
Onycholysis and short distal phalanges of the hands.

**Figure 2:**
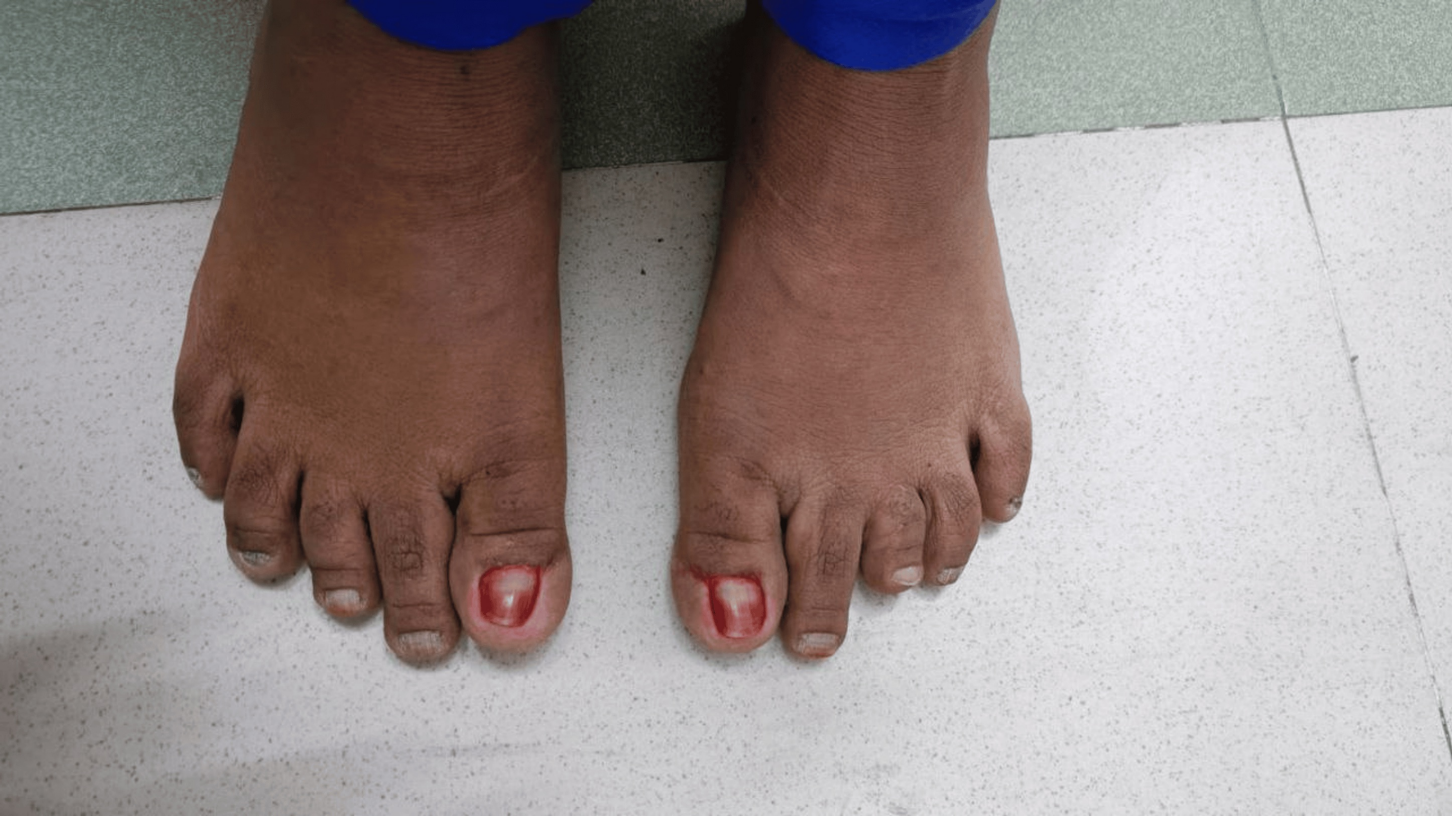
Short left third toe.

On hand examination, the distal phalanges were short, swollen, and appeared clubbed, but there was no obliteration of nail fold angles, considering it as pseudoclubbing. Nails were hyperpigmented prominently in the left hand, and onycholysis was present in the left thumb nail, but there was no nail pitting or transverse ridging (Figure 1). The nail plates were flattened; the width of the nail bed and nail plate was greater than the length. The right wrist joint was mildly tender, and the temperature was not raised. On foot examination, the distal phalanges were noted to be short. The left third toe was shorter with slight pain on palpation of the proximal interphalangeal (PIP) joint (Figure 2). Other systemic examinations were normal.

**Figure 3:**
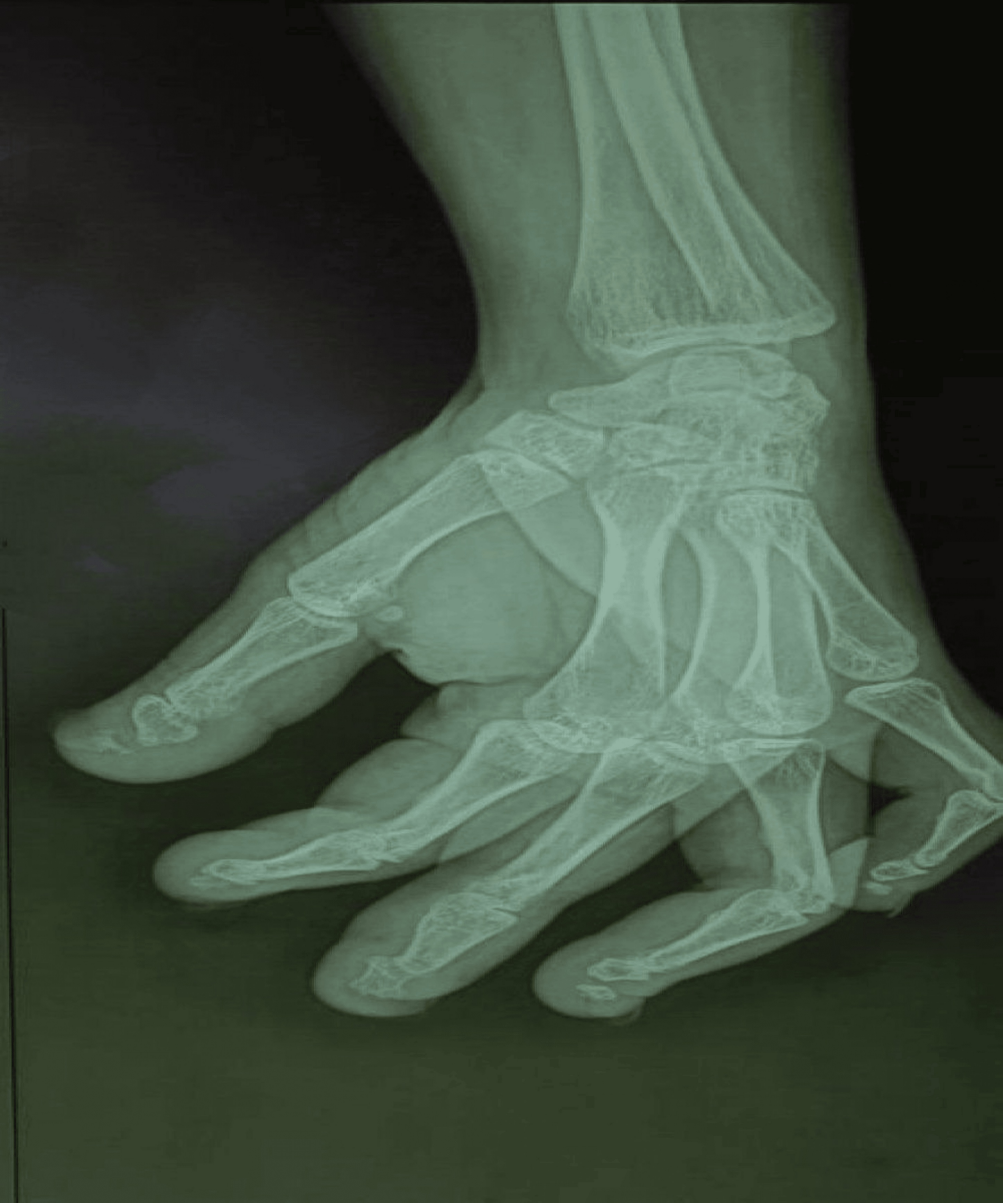
Osteopenia and acro-osteolysis in the hand.

X-ray of the left hand showed osteopenia, acro-osteolysis, and a midshaft fracture of the terminal phalanges (Figure 3). X-ray of the right forearm showed an old healed radial fracture. X-ray of the left foot showed acro-osteolysis and shortening of the proximal phalanx of the left third toe. X-ray of the dorso-lumber spine showed osteopenia, reduced height of vertebral bodies with fish-mouth appearance, vertebral collapse, and kyphosis.

**Figure 4:**
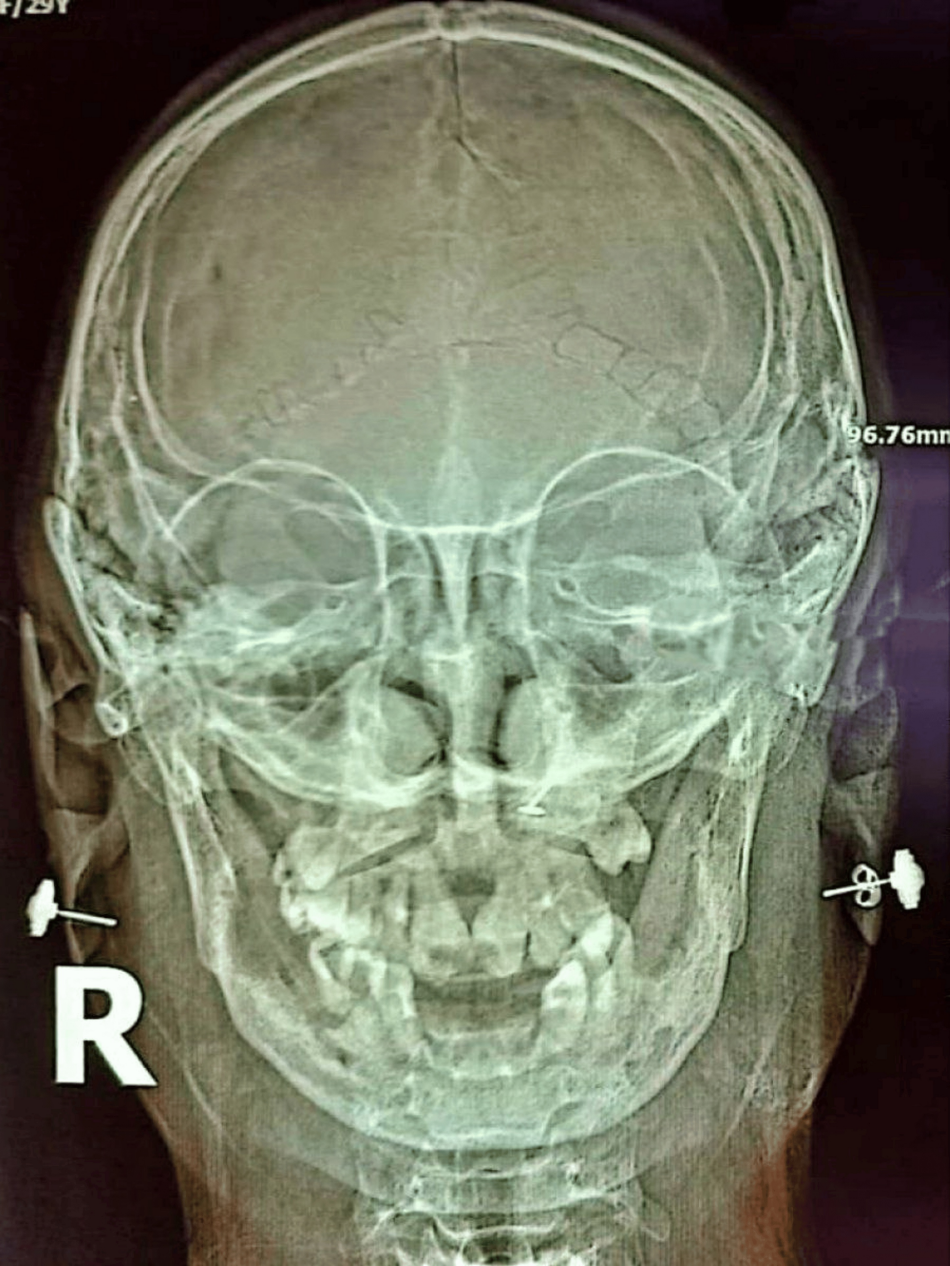
Wormian bones, absence of frontal sinus, and maxillary sinus hypoplasia were noted in the skull X-ray.

**Figure 5:**
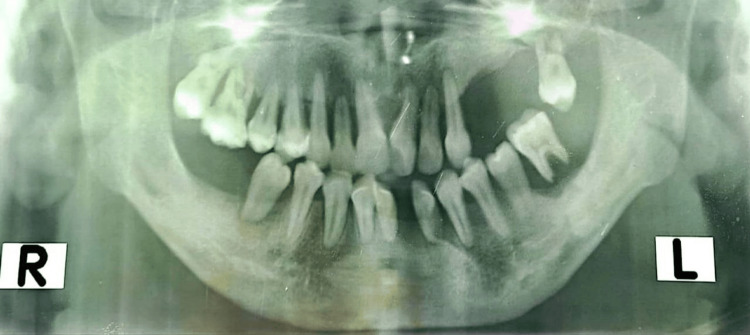
Absence and misalignment of teeth.

X-ray of the skull showed Wormian bones, frontal sinus aplasia, maxillary sinus hypoplasia, and elongated pituitary fossa (Figure 4). X-ray of the mandibles showed the absence of some teeth with misalignment (Figure 5). X-ray of the left leg showed a mild degree of bowing of the fibula.

A series of investigations, including an autoimmune panel, was done to exclude hyperparathyroidism and overlapping connective tissue disease. Her erythrocyte sedimentation rate (ESR) was 29, with a negative rheumatoid factor and antinuclear antibodies (ANA), but a high titer of positive anti-CCP antibodies (>200 u/ml).

The differential diagnosis included other causes of progressive acro-osteolysis and skeletal fragility, such as hyperparathyroidism, scleroderma-associated acro-osteolysis, and other genetic osteolytic syndromes. Hyperparathyroidism was excluded based on biochemical evaluation. The absence of skin thickening, Raynaud’s phenomenon, and visceral fibrosis made scleroderma unlikely. Other inherited skeletal dysplasias were considered; however, the combination of characteristic craniofacial features, dental abnormalities, acro-osteolysis, severe osteoporosis, and a positive family history strongly favored HCS. The presence of inflammatory joint symptoms with positive anti-CCP serology raised consideration of coexisting rheumatoid arthritis, although the atypical joint distribution and preserved mobility suggested a modified or overlapping clinical presentation.

The patient was counseled in detail regarding the nature of her condition, the chronic course of the disease, and the uncertainties surrounding diagnosis and long-term management. Treatment decisions were made with particular caution, taking into account the underlying skeletal fragility associated with HCS and the possibility of coexisting inflammatory arthritis.

Calcium and vitamin D supplementation were initiated to address severe osteoporosis and reduce the risk of further fragility fractures. Bisphosphonate therapy was commenced to improve bone mineral density, recognizing its role in fracture prevention despite limited evidence in rare skeletal dysplasias. Given the presence of persistent inflammatory joint symptoms and strongly positive anti-CCP antibodies, methotrexate was introduced as a disease-modifying agent, with folic acid supplementation. The potential risks and benefits of immunosuppressive therapy were carefully discussed, particularly in the context of severe osteoporosis and atypical joint involvement.

The patient was discharged with a structured follow-up plan involving close clinical monitoring, assessment of treatment response, and surveillance for adverse effects.

This report highlights the need for cautious interpretation of autoimmune findings and tailored therapeutic strategies when managing inflammatory conditions in patients with rare skeletal dysplasias.

## Discussion

HCS is a rare genetic condition that affects connective tissues in the body. It is most commonly inherited in an autosomal dominant pattern, although sporadic cases have also been reported, contributing to diagnostic delay and under-recognition [1].

A key feature of HCS is the degradation of bone tissue (osteolysis), particularly affecting the tips of the fingers and toes (acro-osteolysis) [2]. HCS also presents with unique craniofacial and dental anomalies, short stature, generalized osteoporosis, joint laxity, early loss of teeth, delayed puberty, and impaired vision and hearing [2,3].

Radiological findings in HCS reveal osteolysis affecting the distal phalanges of both hands and feet, an enlarged sella turcica, thickened skull bones, generalized osteoporosis, wide cranial sutures, multiple wormian bones, and an underdeveloped maxilla and mandible with missing frontal sinuses [4]. Our patient exhibited short stature, kyphosis, distinct craniofacial features, missing permanent teeth, and acro-osteolysis. Radiographic findings further supported an HCS diagnosis, showing generalized osteopenia, an enlarged sella turcica, the presence of wormian bones, acro-osteolysis, maxillary sinus hypoplasia, and absence of the frontal sinus [2,3,5-7].

The diagnosis of HCS is primarily based on clinicoradiological pattern recognition, integrating progressive acro-osteolysis with characteristic craniofacial and dental abnormalities, severe osteoporosis with fragility fractures, and supportive imaging findings. A structured diagnostic approach includes a detailed personal and family history, comprehensive clinical examination, and targeted radiological assessment, which together help distinguish HCS from more common acquired causes of osteolysis. Because acro-osteolysis is a non-specific finding, careful consideration of alternative diagnoses is essential. Important differentials include metabolic disorders such as hyperparathyroidism, rheumatologic conditions including systemic sclerosis and psoriatic arthritis, ischemic or neuropathic causes, infectious etiologies, and other inherited osteolytic syndromes [8]. Confirmation of the diagnosis can be achieved through genetic testing for mutations in the NOTCH2 gene, although such testing is not readily available [3,4,7].

In the absence of established evidence for a causal or pathogenetic link, the coexistence of HCS and anti-CCP-positive inflammatory arthropathy in this case is best regarded as hypothesis-generating rather than indicative of a broader association.

Management of HCS remains challenging due to the rarity of the condition and the absence of standardized treatment guidelines. The treatment focuses on addressing the specific symptoms present in each patient, often necessitating a coordinated approach from a team of specialists. Genetic counseling is advised for affected individuals and their families, and providing psychosocial support to the entire family is crucial. Due to the disease's rarity, no large-scale treatment trials have been conducted. Some patients with HCS are treated with bisphosphonates to manage bone resorption and skeletal deformities [5,6].

Limitations

This case report has several limitations that should be acknowledged. It describes the findings from a single patient, and the observations should therefore be interpreted with caution. Genetic confirmation of HCS, including NOTCH2 mutation analysis, was not available, and the diagnosis was based on characteristic clinical and radiological features. The presence of inflammatory joint manifestations in this patient does not allow conclusions regarding a causal relationship or a broader association and should be considered an observational finding. In addition, formal disease activity assessment and long-term follow-up data were limited. Despite these limitations, the case highlights important diagnostic challenges and emphasizes the need for careful clinical evaluation when rare skeletal disorders present with inflammatory joint involvement.

## Conclusions

HCS is a rare skeletal dysplasia that should be considered when progressive acro-osteolysis and marked skeletal fragility occur alongside characteristic craniofacial and dental abnormalities. In this case, the combination of a supportive clinicoradiological phenotype, a positive family history, and exclusion of key mimics strengthened the working diagnosis of HCS despite the lack of genetic confirmation. The co-occurrence of inflammatory joint symptoms with strongly positive anti-CCP serology created a clinically important diagnostic dilemma, highlighting the need to interpret autoimmune markers within the broader clinical and radiological context. Overall, this report adds educational value by outlining a structured diagnostic approach and emphasizing individualized, multidisciplinary management focused on fracture prevention, symptom control, and careful follow-up in a rare and complex presentation.
